# The diversity of clinical Mycobacterium abscessus isolates in morphology, glycopeptidolipids and infection rates in a macrophage model

**DOI:** 10.1099/jmm.0.001869

**Published:** 2024-08-19

**Authors:** Virginia Pichler, Lara Dalkilic, Ghazaleh Shoaib, Tirosh Shapira, Leah Rankine-Wilson, Yves-Marie Boudehen, Joseph D. Chao, Danielle Sexton, Miguel Prieto, Bradley S. Quon, Elitza I. Tocheva, Laurent Kremer, William Hsiao, Yossef Av-Gay

**Affiliations:** 1 Department of Microbiology and Immunology, Life Sciences Institute, University of British Columbia, Vancouver, Canada; 2 INSERM, IRIM, 34293 Montpellier, France; 3 Department of Medicine, University of British Columbia, Vancouver, Canada; 4 Department of Molecular Biology and Biochemistry, Simon Fraser University, Burnaby, Canada

**Keywords:** colony morphology, cystic fibrosis, glycopeptidolipids, *Mycobacterium abscessus*, pathogenesis, host-pathogen interaction, clinical infection, macrophage model, intracellular pathogens, bacterial dissemination, Mycobacteria

## Abstract

**Introduction.** *Mycobacterium abscessus* (MABS) is a pathogenic bacterium that can cause severe lung infections, particularly in individuals with cystic fibrosis. MABS colonies can exhibit either a smooth (S) or rough (R) morphotype, influenced by the presence or absence of glycopeptidolipids (GPLs) on their surface, respectively. Despite the clinical significance of these morphotypes, the relationship between GPL levels, morphotype and the pathogenesis of MABS infections remains poorly understood.

**Gap statement.** The mechanisms and implications of GPL production and morphotypes in clinical MABS infections are unclear. There is a gap in understanding their correlation with infectivity and pathogenicity, particularly in patients with underlying lung disease.

**Aim.** This study aimed to investigate the correlation between MABS morphology, GPL and infectivity by analysing strains from cystic fibrosis patients’ sputum samples.

**Methodology.** MABS was isolated from patient sputum samples and categorized by morphotype, GPL profile and replication rate in macrophages. A high-content ex vivo infection model using THP-1 cells assessed the infectivity of both clinical and laboratory strains.

**Results.** Our findings revealed that around 50 % of isolates displayed mixed morphologies. GPL analysis confirmed a consistent relationship between GPL content and morphotype that was only found in smooth isolates. Across morphotype groups, no differences were observed *in vitro*, yet clinical R strains were observed to replicate at higher levels in the THP-1 infection model. Moreover, the proportion of infected macrophages was notably higher among clinical R strains compared to their S counterparts at 72 h post-infection. Clinical variants also infected THP-1 cells at significantly higher rates compared to laboratory strains, highlighting the limited translatability of lab strain infection data to clinical contexts.

**Conclusion.** Our study confirmed the general correlation between morphotype and GPL levels in smooth strains yet unveiled more variability within morphotype groups than previously recognized, particularly during intracellular infection. As the R morphotype is the highest clinical concern, these findings contribute to the expanding knowledge base surrounding MABS infections, offering insights that can steer diagnostic methodologies and treatment approaches.

## Introduction

Non-tuberculous mycobacteria (NTM) are a class of saprophytic bacteria represented by over 180 species [1]. Ubiquitously found in the environment (soil, water and vegetation), a subset is classified as opportunistic pathogens for their ability to occupy human niches, primarily as pulmonary or soft tissue infections and frequently affecting immunocompromized individuals [1,4]. The most abundant pathogenic species belong to the *Mycobacterium avium* complex (MAC; *Mycobacterium avium, Mycobacterium intracellulare*) and *Mycobacterium abscessus* [MABS; *Mycobacterium abscessus abscessus* (Mab), *Mycobacterium abscessus massiliense* (Mma) and *Mycobacterium abscessus bolletii* (Mbo)], the latter which remains an ongoing treatment challenge due to its intrinsic resistance to growing incidence of broader antibiotic resistance [5,6].

NTM follow a similar pathogenesis strategy, primarily infecting hosts via aerosol inhalation where they interact with and are engulfed by the innate immune cells, namely macrophages, where they survive and replicate in phagocytic vesicles [1,7, 8]. This strategy has been well-documented in MABS infections and is associated with the transition of the bacteria from the smooth (S) to rough (R) morphotype [8]. The difference in intracellular behaviour between the two types has been linked to the presence of glycopeptidolipids (GPLs) [1]. Cell surface GPLs decorate the S morphotype and mask underlying Toll-like receptor 2 (TLR-2) agonists, such as phosphatidyl-myo-inositol dimannoside (PIM2) [9] and lipoproteins [10], and allow for greater initial colonization in loner phagosomes. Conversely, R morphotypes are associated with a strong immunological response, persistent infections and presence in social phagosomes.

**Fig. 1.**
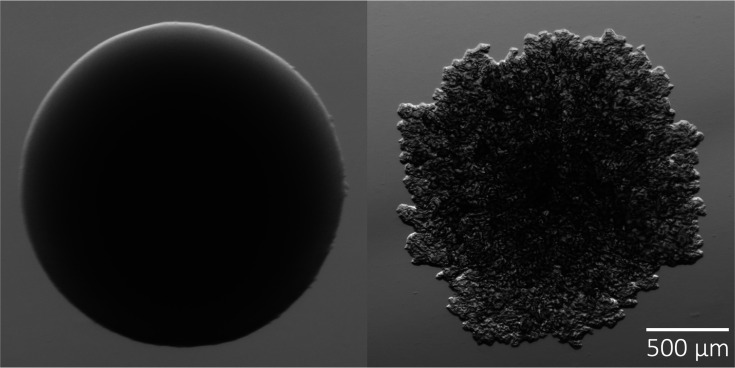
Characteristic two morphotypes of *M. abscessus*: smooth (left) and rough (right). The smooth morphotype is shiny and luxuriant with a uniformly round shape; the rough morphotype is matte, wrinkled and textured with an irregular shape.

The colony morphology of the S morphotype has been described as a uniformly round shape, shiny and luxuriant (Fig. 1, left), whereas the R morphotype is often irregular in shape, matte, wrinkled and textured (Fig. 1, right). The characteristics of these opposite morphologies have, indeed, been widely described in the literature; however, to our knowledge, no deliberate study has been performed to determine a strict set of criteria for each progressive morphotype between the canonical extremes, represented by the S and R laboratory strains ATCC19977/CIP104536 S/R, denoted as Cip^S^ and Cip^R^ throughout. However, rarely do clinical manifestations perfectly mirror their lab counterparts. This is of importance as morphological transition is a marker of infection progression, and establishing these criteria will help reduce inter-observer variability when reporting diagnostic results [11]. Furthermore, following consistent morphological plasticity tracking metrics between clinics and research facilities will help build a more robust pool of data to reveal the full extent of the relationship between morphotype and infection persistence and severity. Here, we describe the isolation, molecular analysis and intracellular growth of 46 clinical isolates from cystic fibrosis patients, represented by an approximately equal distribution of the S and R morphotypes.

## Methods

### Clinical isolates

This study used a total of 46 laboratory-confirmed mycobacterial isolates that were obtained from 19 cystic fibrosis (CF) patients in St. Paul’s Hospital (Vancouver, Canada). Sputum samples were collected, homogenized and inoculated onto various media to identify the bacterial populations present. MABS bacteria were subcultured to ensure that subsequent cultures were pure and free from contamination. Subspecies were identified through matrix-assisted laser desorption/ionization–time-of-flight mass spectrometry (MALDI-TOF MS) and 16s rRNA target sequencing at the B.C. Centre for Disease Control (BCCDC) and subsequently confirmed with whole genome sequencing, with the phylogeny mapped in Fig. S1, available in the online Supplementary Material. Ethical approval was granted by the University of British Columbia (certificate H21-03818).

### DNA extraction and sequencing

Unseparated isolates were grown in 7H9 Broth (Difco Middlebrook) supplemented with 10 % (v/v) OADC (0.05 % oleic acid, 5 % bovine albumin fraction, 2 % dextrose, 0.004 % catalase and 0.8 % sodium chloride solution) and 0.05 % (v/v) Tween 80, termed 7H9OADCT throughout, and placed on a shaker at 37 °C to an OD600 of 1.0–2.0. Genomic DNA (gDNA) was extracted with the cetyltrimethylammonium bromide method, eluted into 50 µl of sterile water and stored at −20 °C.

Samples were transferred to the Genome Sciences Centre (UBC, Vancouver, Canada) for sequencing on the Illumina MiSeq platform (Illumina, San Diego, CA, USA). Each genome was sequenced to a 100-fold depth of coverage. FASTQ files of the paired-end reads were inspected for overall quality using the FastQC programme [12] and assembled using SPAdes v.3.9.0. Assembled genomes were aligned to the reference strain ATCC19977/CIP104536s through conserved genomic regions with Parsnp [21]. Core genome single nucleotide polymorphisms identified from the alignment were then used to construct a phylogenetic tree, which was visualized with FigTree v.1.4.4. FASTQ files were stored on the Centre for Infectious Disease Genomics and One Health’s (Simon Fraser University) Digital Research Alliance of Canada computing cluster.

### Strain separation

BCCDC glycerol stocks were streaked onto Luria–Bertani (LB) agar plates and incubated at 37 °C for 4–6 days. Isolates were visually inspected for morphotype composition at high resolution with the Zeiss Axio Zoom V16 microscope according to the categorical descriptors in Table 1. Plates containing heterogenous populations were re-streaked from single colonies until pure, stable morphotypes were achieved. Single colonies were inoculated in 7H9OADCT and grown to mid-log phase for glycerol stocks (stored at −80 °C).

**Table 1.** Canonical features of smooth (Cip^S^) and rough (Cip^R^) morphotypes

	Cip^S^	Cip^R^
Appearance	Shiny	Matte, textured
Colour	Brown	Grey
Margin	Uniform	Irregular
Glycopeptidolipid	Present	Absent
Infection stage	Colonizing	Persistent
Antibiotic resistance	Low	High

### Lipid extraction

Fresh bacterial lawns on LB agar were harvested and pelleted in glass vials by centrifugation at 2000 g for 5 min. Polar glycopeptidolipids were extracted using the chloroform-methanol method, as described previously [13]. Final extracts were solubilized in 300 µL of chloroform/methanol (2 : 1 v/v). Lipids were visualized with thin layer chromatography (TLC) by adding 20 µL of sample on a silica gel 60 F254 sheet (Merck) and placed in a chloroform/methanol/water (90 : 10 : 1, v/v/v) solution for migration. An orcinol-sulfuric acid solution (0.2 and 20 %, respectively) was sprayed onto the sheet, and the plates were lipid profiles revealed using heat. GPLs were detected with reference to the control strains [Cip^S^, CIP104536T (S) and Cip^R^, CIP104536T (R)]. Replicates were performed for strains where morphotype categorization did not align with the TLC results.

### Ex vivo THP-1 macrophage infection model

#### Construction of an integrative vector expressing a fluorescent marker

Derivative versions of the integrative plasmid pMV306 [14,15] were constructed to allow strong expression of either mScarlet (pMV306-mScarlet, Fig. S2) or mWasabi (pMV306-mWasabi) fluorescent proteins with a kanamycin-resistant cassette for selection. This plasmid contains pMV306 integrase *attP* as a one-step integration construct. Briefly, the mScarlet or mWasabi coding sequences, placed under the control of the constitutive P left* promoter, were PCR-amplified by Q5 High-Fidelity DNA Polymerase (New England Biolabs) using plasmid L5 attB::Pleft*mScarlet/mWasabi (Addgene plasmids 169 410 and 169 409, respectively) as DNA templates [16]. Primer pairs are as follows: dual forward primer P*: ATCTTTAAATCTAGATGGCCGCGGTACCAGATCTT; mScarlet reverse primer: AGCTGGATCCATGGATTCACTTGTACAGCTCGTCCATGCC; mWasabi reverse primer: AGCTGGATCCATGGATTTACTTGTACAGCTCGTCCATGCC. The linear fragments were purified on agarose gels (NucleoSpin Gel and PCR Clean-up, Macherey-Nagel). Following the manufacturer’s instructions, Pro Ligation-Free Cloning Kit (abm) reactions were performed to insert these linear fragments into the pMV306 previously digested with *Escherichia coli* strain RV and transformed into *Escherichia coli *Stellar TM competent cells (Takara Bio), purified (NucleoSpin Plasmid, Macherey-Nagel) and verified by DNA sequencing. Plasmids were then electroporated into the *M. abscessus* strains [17,18], and recombinant clones harbouring the constructs inserted at the attL5 insertion site in the *glyV tRNA* gene were selected for 7H10 agar supplemented with 0.5 % glycerol, 10 % OADC and 50 µg ml^−1^ kanamycin (7H10OADCKan).

### Bacterial and THP-1 cell culturing

Unless otherwise stated, all bacterial strains were transformed with pMV306-mScarlet and routinely grown from stock at 37 °C in 7H9OADCT with 50 µg ml^−1^ kanamycin, termed 7H9OADCTKan. THP-1 human monocyte-derived macrophage-like cells (ATCC TIB-202) were grown in incomplete Roswell Park Memorial Institute (RPMI1640) medium (10 % FBS, 2 % glutamine and 1 % non-essential amino acids) at 37 °C with 5 % carbon dioxide (CO_2_). Cell density was kept between 0.25 and 1×10^6^ cells ml^−1^ for a maximum of 3 months.

### Infection

THP-1 cell suspension at 5×10^5^ cells ml^−1^ in incomplete RPMI1640 was seeded into a 96-well plate at 50 000 cells/well and differentiated into a macrophage-like state over a period of 48 h with phorbol12-myristate13-acetate (40 ng ml^−1^) at 37 °C with 5 % CO_2_. Liquid bacterial cultures were grown to mid-log phase in 100 µl per well in a round-bottomed 96-well plate (VWR), centrifuged (5000*g*, 10 min) and washed once with sterile Dulbecco’s phosphate-buffered saline (DPBS, Gibco). Cultures were de-clumped using a 25G blunt needle and transferred to a flat-bottomed 96-well plate (VWR), and OD_600_ was measured (OD_600_ of 1 ≈ 1.13×10^9^ c.f.u. ml^−1^) using the Varioskan LUX microplate reader (Thermo Fisher Scientific). Inoculum of a multiplicity of infection (MOI) of 2 bacteria per macrophage was prepared in incomplete RPMI1640 and opsonized with 10 % non-decomplemented human serum for 30 min at 37 °C, shaking. Differentiated macrophages were washed once with warm DPBS and inoculated with the opsonized bacterial suspensions for 3 h (37 °C, 5 % CO_2_). Wells were washed twice with DPBS and treated with amikacin (250 µg ml^−1^) for 1 h to remove any extracellular bacteria. The amikacin was removed, and wells were washed a final time with DPBS and replaced with 100 µl incomplete RPMI containing 1 µg ml^−1^ Hoechst 33 342. Plates were incubated for 3 days at 5 % CO_2_ at 37 °C. Testing of each strain was performed in technical triplicates.

### High-content intracellular growth analysis

Intracellular bacterial growth was monitored using the CellInsight™ CX5 High Content Platform (Thermo Fisher Scientific) using methods previously described by our group [19,20]. Briefly, macrophages were identified and counted through nuclei staining, and a mask was created to represent the entire cell, or region of interest (ROI/circle). Red channel fluorescence (569/593 nm) was used to detect bacteria ‘spots’ inside the cellular ROI. These spots were quantified using a variety of measurements including intensity and area using the Thermo Fisher Scientific™ HCS Studio™ Cell Analysis Software. These fluorescent measurements closely correspond with colony-forming units (c.f.u.), as previously validated [12]. Data were plotted using GraphPad Prism version 10 (GraphPad Software, Boston, Massachusetts, USA, www.graphpad.com). Measurements were captured at multiple time points to monitor replicate rate and bacterial burden. Super-resolution fluorescence light microscopy of the 62S and 62R isolates expressing mScarlet was used to determine morphotype differences after 72 h of infection: macrophages were differentiated onto coverslips, and infection was carried out as described above, after which cells were fixed with 4 % paraformaldehyde and imaged using Zeiss LSM 900 confocal microscope equipped with an Airyscan 2 detector and a Colibri 5 light source. Images were collected with a Plan-Apochromat 100×/1.46 oil objective lens and processed using Zen Blue 2.4 software.

## Results

### Subspecies distribution in CF isolates is predominantly Mab

Nineteen patients provided 46 individual sputum samples, which were subcultured and speciated using MALDI-TOF MS and whole genome sequencing. Subspecies representation was dominated by Mab in categories such as prevalence within patients (*n*=14, 73.68 %) and total clinical isolates (*n*=38, 82.61 %) (Table 2). Mma was the next most prevalent, with the subspecies isolated in 5 of the 19 patients (26.32 %). Mbo was isolated from the single sputum sample provided by patient 16. S and R morphotypes were evenly distributed amongst patients, isolates and strains. Sixteen patients returned more than one morphotype in their sputum samples, and of the 46 BCCDC isolates collected, 27 (58.7 %) were further separated into both defined S and R morphotypes. All BCCDC isolates were correctly identified to the subspecies level and phylogenetically mapped using whole genome sequencing data (Fig. S1).

**Table 2.** Patient sample isolation dates, species and morphotype categorization

Patient (*n*=19)	Isolate (*n*=46)	Isolation date (YYYY-MM-DD)	Species		Morphotypes	
				S (*n*=37)	R (*n*=35)	‘R-’ (*n*=3)
1	10	2013-03-07	Mab	•		
	F	2018-06-08	Mab	•	•	
	77	2018-08-15	Mab	•	•	
	M	2019-01-17	Mab	•	•	
	K	2020-02-03	Mab	•	•	
	J	2021-05-05	Mab	•	•	
	G	2021-05-05	Mab		•	
						
2	8	2013-06-24	Mab	•	•	
	26	2014-03-09	Mab		•	
	45	2015-07-12	Mab	•	•	
	49	2016-03-18	Mab	•	•	
	D	2016-10-05	Mab		•	
	I	2017-06-09	Mab		•	
						
3	79	2018-07-09	Mab	•		
	76	2018-08-13	Mab	•		
	C	2018-08-13	Mma	•		
	B	2018-12-01	Mab	•		
						
4	54	2016-06-28	Mab	•	•	
	57	2016-10-21	Mab	•	•	
	L	2019-05-09	Mab	•	•	
	A	2019-09-19	Mab	•	•	
						
5	30	2014-12-01	Mab	•	•	
	43	2015-05-10	Mab		•	
	52	2016-06-20	Mab	•	•	
	62	2017-01-19	Mab	•	•	
						
6	E	2018-10-29	Mab	•	•	
	81	2018-10-29	Mab	•	•	
						
7	50	2016-03-31	Mma	•		
	82	2018-05-12	Mma	•	•	•
						
8	29	2014-11-20	Mab	•		
	42	2015-09-21	Mab	•	•	
						
9	27	2014-02-10	Mab	•		
	72	2018-10-01	Mab	•	•	
						
10	70	2017-10-15	Mma		•	
	80	2018-09-27	Mma	•	•	•
						
11	22	2014-04-30	Mab	•		
	N	2019-01-23	Mab		•	
						
12	6	2013-04-18	Mab	•	•	
						
13	7	2013-05-28	Mab		•	
	15	2013-11-29	Mab	•	•	
						
14	16	2013-05-12	Mma	•		
						
15	23	2014-01-06	Mab		•	
						
16	25	2014-03-09	Mbo	•	•	
						
17	67	2017-09-07	Mma	•		•
						
18	75	2018-08-09	Mab	•	•	
						
19	H	2019-05-22	Mab	•	•	

gDNA was initially extracted from the unseparated clinical isolates. As separation of morphotypes became more relevant to a larger, overarching study, examining the total 75 individually characterized clinical strains by morphology is underway and will be described once completed. Analysis of the unseparated gDNA showed that serial isolates from the same patient generally clustered together phylogenetically (Fig. S1), suggesting a consistent infection rather than co-infections or re-infections over time. Notably, serial isolates from Patients 2, 8 and 13 displayed phylogenetic discrepancies, clustering into different clades. Isolates with alphabetic identifiers are absent from the phylogenetic tree, as they were from sputum samples collected during the course of the study and thus received after sequencing was undertaken.

### Colony morphology variation among MABS clinical isolates

Colonies were isolated and subcultured several passages to ensure that pure morphotypes were reproducible and stable. Strain colonies were inspected using high-resolution zoom microscopy and evaluated for morphotype composition. Colonies were most often presented as having uniform margins and smooth texture (‘S’, Fig. 1, left) or irregular margins with rough texture throughout (‘R’, Fig. 1, right) and were classified accordingly.

### Visual subcategorization of smooth-like colonies lacks reproducibility

**Fig. 2.**
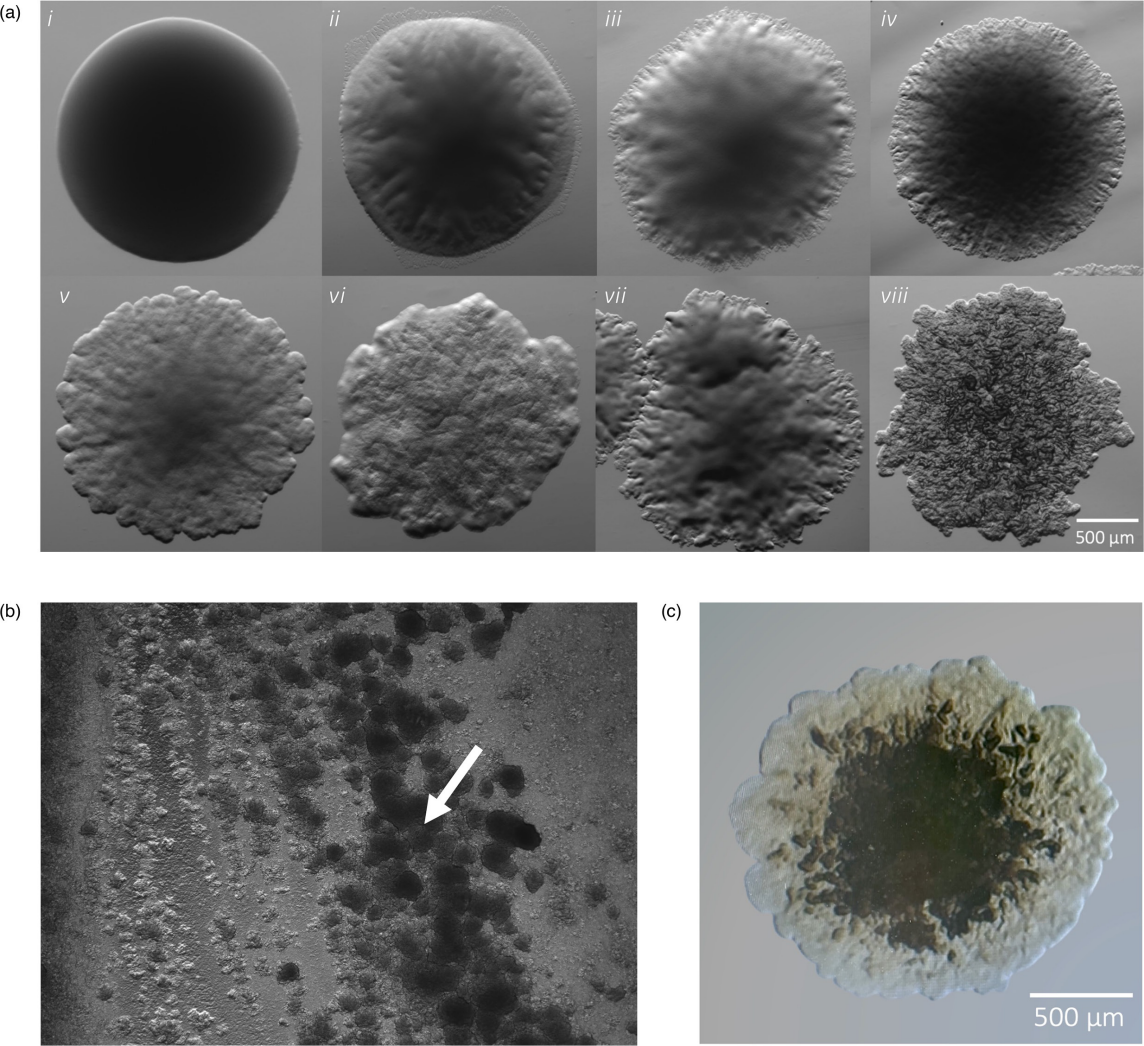
(a) *M. abscessus* morphology variability. Several ‘intermediates’ colony subtypes (ii–vii) appear among clinical strains and deviate from the strict S or R morphotype, represented by the first and last images in the series. (b) The putative ‘R-’ phenotype appears rough in individual colonies but adopts a smooth-like characteristic when plated densely (white arrow). (c) Putative ‘R-’ colony with rugged edges, smoother centre and brown colour.

In addition to the canonical morphotypes, several strains appeared as ‘intermediates’, along a spectrum between smooth and rough (Fig. 2a). A sub-morphotype classification system was initially used to categorize these intermediates, by way of ‘S-’, ‘S- -’ and ‘R-’, where S- (Fig. 2a and ii–iv) is more similar to the reference S morphotype (Fig. 2a(i)), than S- -, which displays further edge ruffling and is more matte in appearance (Fig. 2a(v–vi)). This categorization strategy became unwieldy, in that there was no definitive characteristic that would separate any one subcategory from their neighbour, and the variability of shape and texture extended beyond these three discrete groupings. In addition, the intermediate colony morphology strains were often not stably reproducible after repeated subculturing. This phenomenon was observed with both intermediate transitioning to smooth, and *vice versa* over time. We did not observe nor subsequently explicitly test for morphotype transition to true rough and later recategorized S- and S- - into the recognized S morphotype class.

### Some Mma isolates exhibit density-dependent intermediate morphology

The putative ‘R-’ category was defined as individual colonies that consistently carried rough-like features, yet, when plated densely, it would harbour a central, smooth-like phenotype with rough edges (Fig. 2b, c). This phenotype was observed in three patients’ unique sputum samples and always belonged to Mma subspecies. Twelve (16 %) of the 75 strains belonged to Mma. Patient 10 provided two individual samples, 1 year apart, both only containing Mma (Table 2). The first sample yielded a strict R morphotype Mma (70R), while the second sample contained all three morphotypes (S=80S, R=80R and R-=80R-). To further characterize these morphotypes, we next experimentally assessed lipid composition in each strain to determine the presence of GPL.

### GPL lipid profiles correlate with colony morphology in Mab more than Mma

**Fig. 3.**
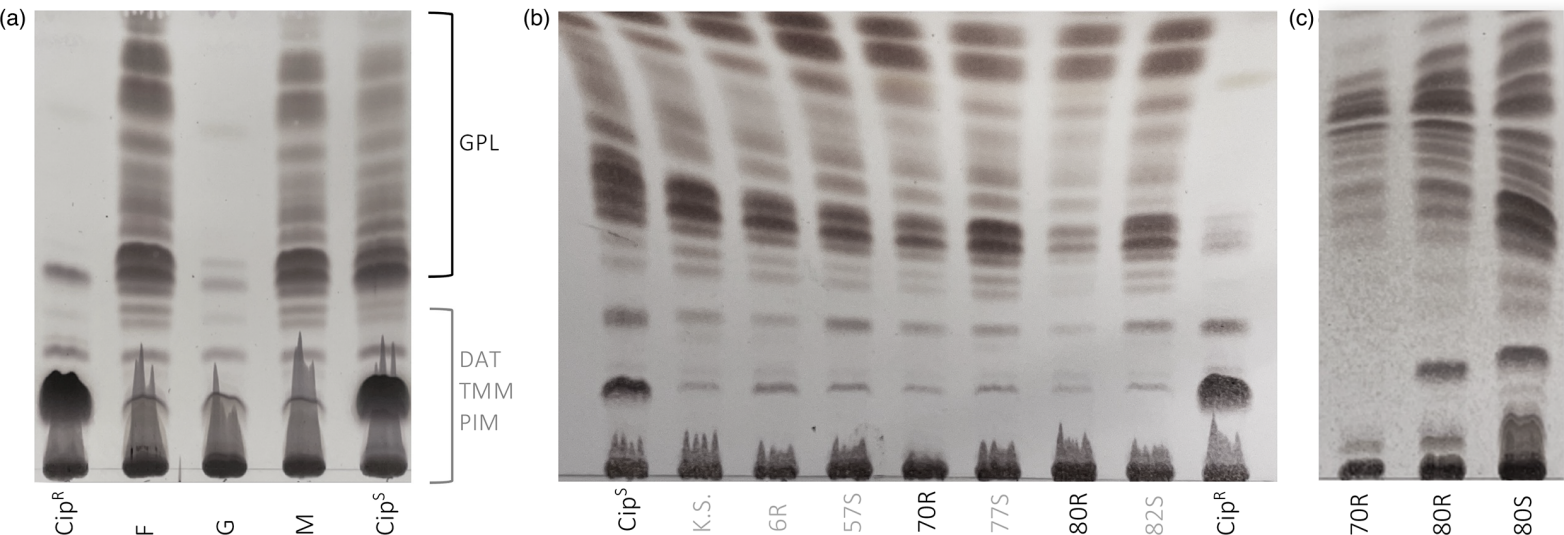
GPL profiles. (a) Cell surface GPLs are shown in the upper banding of the TLC, with diacyltrehalose (DAT), trehalose monomycolate (TMM) and phosphatidylmyo-inositol mannoside (PIM) lipids making up the lower bands. GPLs present in isolates F and M are S morphotypes and match the associated lab strain (Cip^S^); isolate G is an R morphotype and matches its associated lab strain (Cip^R^). GPL will be present on TLC of mixed morphology isolates, as the R morphotype is confirmed only by the absence of GPL. Mixed isolates should first be subcultured into pure morphotypes before polar lipid extraction and visualized on a TLC. (b) TLC of GPL composition in several clinical isolates, already separated by morphotype. 70R and 80R/R- were morphologically categorized as rough strains yet present with GPL banding. Isolates 70 and 80 are serial collections from the same patient and belong to the subspecies Mma, which may indicate morphological features unique to the subspecies that differ from Mab. (c) Repeat extraction and TLC of isolates 70 and 80 to confirm GPL presence.

GPL presence was qualitatively assessed using TLC of lipid extracts. This assay is a simple way to score strains as GPL-positive or -negative by the presence or absence of banding, respectively (Fig. 3a). GPL presence and strain morphotype were correctly matched in 100 % Mbo, all S-typed strains and ~97 % of Mab isolates (unmatched *n*=1, rough strain J (J.R.) in Patient 1). We recorded five patients who provided samples that contained Mma, which were categorized by BCCDC into seven (15.2 %) isolates that we further classified into 12 distinct strains. As previously stated, three of these samples were identified as putative R- morphotype (67R-, 80R- and 82R-), all of which displayed clear banding in the GLP region of the TLC assay, indicating presence of GPLs (Fig. 3b, c). Retrospectively, all 12 strains would belong to the S morphotype group when considering GPL production.

The strains were subcultured into both solid and liquid media to ensure that purity and lipids re-extracted a total of three times, each instance generating consistent results. Furthermore, the GPL profile to morphotype inconsistency was observed in three Mma strains that were grouped as strict R (70R, 80R and 82R). Mma strains 67, 80 and 82 also possessed strict S morphotypes, showing that Mma has more than one morphology associated with the presence of GPL. This leads us to question whether colony morphology should be supported as a method of morphotyping in Mma strains and further highlights the inconsistency with this method across all MABS species. We report that the GPL production of Mma strains is not readily determined by visual inspection of colony morphology.

### High-content evaluation reveals intracellular replication is the highest among clinical R strains

A THP-1 macrophage infection model was used to better approximate the behaviour of the separated strains in the context of host. A total of 69 strains, including reference strains, were successfully electrotransformed with the pMV306-mScarlet integrative plasmid containing the red fluorescent protein, which was used to identify the intracellular bacterial burden over time. Relative fluorescence from the transformed bacteria was tracked over 3 days to assess intracellular replication rate. All fluorescence values were normalized to the first time point at 4 h to account for any variations in the MOI. Intracellular bacterial burden was evaluated using several parameters, including the relative replication rate of each strain to its respective lab strain, the degree of variance between replicates and the comparison of each morphotype by collapsing data from all strains into S, R or R- categories.

### Growth characteristic of the R- subtype is more closely related to S morphotype than R

**Fig. 4.**
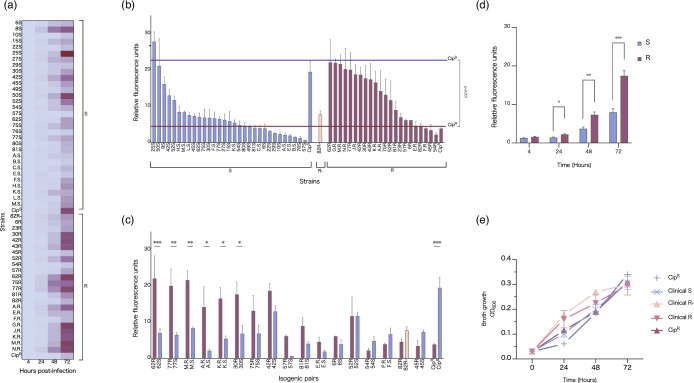
Clinical strain growth rates. (a) Relative fluorescence intensity of intracellular *M. abscessus* clinical strains at 24, 48 and 72 h, as normalized to the 4 h starting time point. All strains were prepared to the same starting inoculum concentration and left to infect THP-1 macrophages for 3 h in triplicate, followed by a 1 h 250 µg ml^−1^ amikacin treatment to clear any remaining extracellular bacteria. Lower fluorescence intensity is represented in pale green and high intensity in dark blue. Broadly, S strains (left of plot) replicate as a slower rate than the R strains (right of plot), with the greater divergence appearing at 48 and 72 h. Reference strains Cip^S^ and Cip^R^ adopt the inverse replication rate to their clinical counterparts. Overall, there is greater replication rate variability among the R strains. Isolate 82R- was classified as an ‘intermediate’ morphotype clusters more closely with the S strains. Other notable outliers, including 25S and 50S, belong to the subspecies Mbo and Mma, respectively. (b) Intracellular bacterial growth of all isolates at 72 h post-infection, grouped by variant. (c). Intracellular bacterial growth of isogenic pairs at 72 h post-infection. A one-way ANOVA showed statistical significance between the lab strains, Cip^S^ and Cip^R^ (*P*=0.0016), and additionally between the isogenic pairs of 62 (*P*=0.009), 77 (*P*=0.038) and M (*P*=0.0052). (d) All strains collapsed into the morphotype group over 72 h. There were no significant differences in rate between groups (S, *n*=37; R, *n*=35; R-, *n*=3). (e) Clinical isolate intracellular bacterial replication measured through fluorescence intensity, collapsed by morphotype (S, *n*=31; R *n*=22; R-, *n*=1). R strains replicate at a rate approximately 2-fold higher than that of the S strains and differ significantly at 24, 48 and 72 h p.i. The ‘R-’ intermediate strain (*n*=1) significantly differed from the R strains, but not the S, indicating it clusters with the intracellular replication behaviour of the S morphotype.

To evaluate each clinical strain relative to its respective lab strain (Cip^R^ or Cip^S^), a multiple comparison test was performed with a one-way ANOVA on relative fluorescence captured at multiple time points (Fig. 4a and Table S1) and presented as individual, isogenic pairwise and grouped (Fig. 4b–d, respectively). Cip^S^ replicated at a rate approximately sixfold greater than Cip^R^ and was found to be statistically significant (*P*<0.001). Interestingly, clinical strains replicated faster than their cognate lab strain, a characteristic not often reported in clinical strain research. When grouped as isogenic pairs, faster growth was also observed in most R strains compared to S strains in the macrophage model (Fig. 4c). Indeed, when looking at isogenic strain pairs, nearly all R strains replicated about twice as fast as their S counterparts. Of these, isolates 62, 77, M, A, K and 30 were found to be significantly different. Isolates 77, M and K are sequential collections from Patient 1 and possess both S and R morphotypes. These strains had similar replication rates which were significantly greater (*P*<0.01) than that of the isolate (F.R.) collected 2 months prior, indicating a degree of Mab population stability over the subsequent 18 months of infection (Table 2). Isolates 30 and 62 were the bookends of serial samples provided by Patient 5 for this study and also returned significance between the S and R replication rates, though no significant differences between the other isolates of Patient 5 were found. The replication rate of *in vitro* growth was also measured by OD_600_ and revealed negligible differences between S and R morphotypes, after 72 h (Fig. 4e).

To investigate morphotypes as a group, all fluorescence data were collapsed into the discrete S, R and R- clusters to evaluate for broader trends, not otherwise discernible from individual pairs (Fig. 4d). All R strains collectively showed a significant twofold growth to the S strains at 24, 48 and 72 h showing that intracellular growth is indeed higher in R than S. The putative R- strain closely matched the replicative behaviour of the smooth strains and replicated at a significantly slower rate compared to the bona fide R group.

Considering the faster growth of clinical isolates seen in this study, we wanted to see if variance was affected to a greater extent in clinical strains, and if so, which morphotypes contribute to such variation? We performed a Levene’s test to evaluate (i) the degree of variance between replicates within a single strain and (ii) if any significant differences existed between strains (data not shown). This test was selected as it does not assume a normally distributed population, allowing for the use of different measures of central tendency to calculate deviations from the group centre, making it robust for data that might be skewed or have outliers (as with clinical samples). As expected, variance was greatest at 72 h across all strains, although no significant differences were observed between strains (*P*>0.05).

### Reference strains Cip^S^ and Cip^R^ infect macrophages at different rates compared to their clinical counterparts

**Fig. 5.**
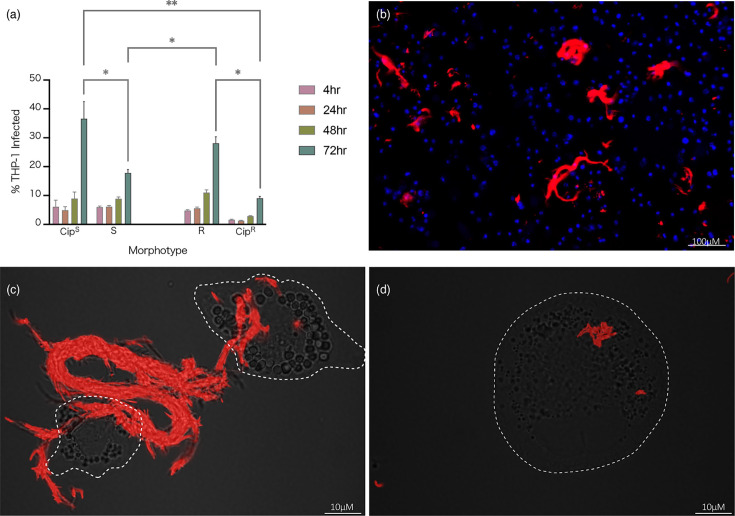
Infection burden. (a) Per cent of THP-1 macrophage infection by morphotype, clinical and lab strain. The proportion of infected THP-1 macrophages was significantly greater among the R clinical strains compared to their S counterparts at 72 h post-infection (** indicates P = 0.0074 and * indicates P < 0.02). Clinical morphotype groups also infected a greater percentage of THP-1 macrophages compared to their associated lab strains. (b) High-content fluorescent microscopy (CellInsight™ CX5 High Content Platform, Thermo Fisher Scientific) image of isolate 62R at 72 h post-infection. Macrophage nuclei are stained blue, and MABS is expressing mScarlet, with notable cording forming extracellularly. (c, d) Super-resolution fluorescence light microscopy of 62R (**d**) and 62S (**e**) isolates, 72 h post-infection. The R morphotype is found on the surface of macrophages (outlined with white dashed line) with distinct ‘cording’ appearance indicative of cell aggregates too large for clearance by phagocytosis.

The proportion of infected THP-1 macrophages was calculated as a percentage of macrophages with intracellular MABS burden over the total macrophage population per condition (Fig. 5a). Cip^S^ infected a significantly greater proportion of cells compared to the clinical S strains. The clinical R strains (*n*=22) infected a greater percentage of macrophages, both compared to the lab strain (Cip^R^) and the clinical S strains (*n*=31) at 72 h post-infection (*P*<0.05). The high replication rate of Cip^S^ compared to the clinical S strains complements the higher proportion of macrophages infected. Clinical R strains replicated faster and infected a greater proportion of infected macrophages compared to the Cip^R^, whereas inversely, clinical S strains replicated slower and a lesser proportion of infected macrophages compared to Cip^S^. This highlights a divergence in pathogenesis of the clinical strains from their associate lab strains. No significant loss in THP-1 survival was seen over the course of infection, either between morphotypes or strains. Further microscopic analysis of the variants revealed that, indeed, a feature of the R morphotype at later time points is the formation of extensive extracellular cording, exceeding the size of macrophages and hampering phagocytosis as a clearance strategy (Fig. 5b–d).

## Discussion

This study’s aim to characterize S and R morphotypes of *M. abscessus* clinical infections was initiated with the separation of strains into the two discrete groups. Strict S colonies are characterized by a uniformly round shape, a smooth margin, an absence of interior texture and a beige or brown colour, while strict R colonies are characterized by an irregular shape, rough margins, an interior texture and a grey colour (Table 1 and Fig. 1). After multiple passages of each strain to attain sample purity, it became evident that these two morphotype classes were insufficient to capture the full morphological spectrum.

Subculturing methods aim to isolate a single, pure, stable sample with only one morphotype present. We found some isolates readily conformed to a stable morphotype, but some were highly variable over more than five passages. It is important to note that subcultures with mixed populations never generated both strict S and R morphotypes on the same plate but rather contained several sub-morphotypes appearing as ‘intermediates’ between S and R. Initially, these intermediates were classified as ‘S+’, ‘S++’ and ‘R-’ and fell serially between S and R (i.e., S, S-, S- -, R- and R). A subculture isolated from a strict S colony may yield S, S+ and S++ on the same plate, while other strains would only yield more strict S colonies. While the intermediates continued to appear in the subcultures, the inability to successfully isolate consistent sub-morphotypes indicates morphological variations as transient features. Conditions that may have affected colony morphology, such as density, culture age and media type, likely contributed to the observed variability; however, a separate sub-study would need to be performed to properly characterize the full range of factors involved, as has been done on *Pseudomonas aeruginosa* [11]. Despite this limitation, all strains were separated and classified broadly as S and R; all S sub-morphotypes (S+ and S++) fell under the umbrella of S while all strains classified as R adopted the strict morphotype. The ‘R-’ sub-morphotype, while appearing most like the R morphotype, presented with less texture and brown, rather than grey, in colour (Fig. 2c). Additionally, this sub-morphotype forms concentrated, smoothed patches when plated as a lawn (Fig. 2b), a feature absent in the strict R morphotype, which is consistently sharply textured even when plated to lawn density. Notably, when reviewing the associated clinical metadata, all strains that possessed the R- sub-morphotype belonged to a congruent subspecies, Mma, and were observed in three of the total seven isolates. While these findings have revealed a common feature of this subspecies’ colony behaviour, the R- sub-morphotype was (1) found in mixed populations, often among strict S and R colonies, and (2) not ubiquitous across all seven Mma strains and therefore cannot serve as a stable speciation proxy for all Mma isolates with microscopy.

Clinical *M. abscessus* research has predominantly been performed *in vitro*, which does not adequately reproduce the complexity of the host environment and immunological responses to infection [21]. As the presence of GPL is an important factor in *M. abscessus* pathogenesis, evaluating these bacteria as they interact with macrophages provides a better opportunity to investigate the contributing role of GPL during infection. For *M. abscessus*, a knowledge gap that exists regarding the heterogeneity of a bacterial population and their dynamics during infection may be resolved using high-content analysis. When collapsed by morphotype, we found no significant differences in *in vitro* growth data between groups (Fig. 4e). A THP-1 macrophage infection model was then used to better approximate behaviour ex vivo. We found inverse relationships between clinical and reference morphotyped strains in their ability to infect a population of THP-1 cells over time (Fig. 5a). We also noted that clinical R strains grew faster than Cip^R^ in our infection model, a trait that reversed in S morphotypes (Fig. 4b). Employing three technical replicates within each experiment was chosen to balance precision and resource efficiency, given that the 54 strains broadly fell into two categories.

Traditional c.f.u./millilitre assays include a consistent amikacin treatment to control for extracellular growth, thus ensuring that colony quantification post-lysis exclusively comes from the intracellular environment; however, this ignores the process of bacterial escape from macrophage lysis. MABS infection persistence is a combination of two strategies: (1) intracellular survival and apoptosis suppression by the S morphotype and (2) bacterial escape and extracellular aggregate colonization (cording) by the R morphotype (Fig. 5b–d) [2,8]. By removing the amikacin treatment step over the course of the infection, a truer reflection of infection burden could be seen due to free movement of the bacteria within and between macrophages. Capturing the increase in per cent of infected cells demonstrates a degree of bacterial escape and new macrophage infection. Our results show that at 48 and 72 h post-infection, the per cent of infected macrophages increased in both morphotypes, clinical and lab strains alike. One limitation must be addressed in that our pool of clinical R strains, including most R- strains, was depleted due to difficulty with mScarlet transformation. Some strains underwent several transformation attempts, with additional cell wall perforation methods to enhance DNA uptake [18]. Transformation of mycobacterial species can be especially challenging due to the complex composition of in the cell wall, which often hampers uptake of foreign DNA. The abundance of lipids in the cell wall additionally renders the bacilli hydrophobicity, resulting in clumping when grown in media and necessitating the use of de-clumping methods during the preparation of electrocompetent single cells. R morphotype strains have been noted as the most challenging to transform and most often form clumps [2]. This was the case for these clinical strains, with ~88 % (*n*=7) of those unsuccessful belonging to the R morphotype.

Nevertheless, the greatest increase observed in infected macrophages was observed in the Cip^S^, clinical R, clinical S and, finally, Cip^R^, reaffirming that the clinical strains do not predictably follow the behaviour of their associated lab strains. Identifying this divergence holds broad implications for our understanding of MABS pathogenesis and persistence. Many studies [2,7, 22,24] have delved into the importance of morphotype distinction, their unique intracellular behaviours and their strategies to successfully persist in the host environment, but our findings highlight that the features observed in lab strains may be insufficient to fully appreciate the complete diversity and variability found in clinical strains. Most infection studies to date have been completed using reference strains (ATCC199977/CIP104536S/R) alone or fewer than ten clinical isolates, limiting the translatability of the results to authentic clinical contexts [2,24,27]. This seems to be especially salient as not only do our results show the breadth of behaviour across the strains during infection but show that clinical morphotypes broadly behave inversely to their lab counterparts.

While the overall trend showed clinical R strains replicating at higher rates than the S strains, large ranges were still observed within each morphotype, with some of the strains more closely matching their associated lab strain. Isogenic strains also varied by fold-difference after 72 h. Eighty three per cent of R strains replicated faster than their S counterparts, with 75 % of the faster growing R strains replicating at 2- to 3-fold greater than their S strain pair. Of the pairs that saw S strain outgrow R, we highlight the profiles of Patients 1 and 4, where earlier isolates (F and 54, respectively) showed slower replication in the R versus S. However, this trait was reversed in the isogenic pairs from the next patient collection (77 and 57, respectively). In both instances, this was accompanied by the decline in lung function capacity (data not shown). R colony density and shape variability has been noted by Park *et al*. [28] in the context of progressive disease; however, the full phenotype spectrum has yet to be elucidated, as is evident by our findings of varied intracellular replication rate and other data describing mutational and transcriptional changes [27,29,31]. Strains 25S and 50S displayed the greatest amount of intracellular growth within their morphotype at 72 h p.i.; however, these two strains belong to the subspecies Mma and Mbo, respectively, which could account for notable difference.

The higher proportion of infected cells seen in clinical R morphotype may be due to bacterial escape and subsequent increased macrophage TLR receptor engagement to immunostimulatory surface molecules, such as PIMs and lipoproteins, that are otherwise masked by GPL in the S morphotype [32]. The stable cell survival between both morphotypes and increase in infected cells over time do not support the supposition of macrophage apoptosis and bacterial release. The increase in infected macrophages without cell lysis may attributed in part, to the stimulation of, membrane channels that connect cells as a rescue function during stress for the transfer of essential cellular components, such as mitochondria [33]. TNT-like structures have been shown to play a role in dissemination of several intracellular pathogens and have been observed in other mycobacteria, including *Mycobacterium marinum* and *Mycobacterium bovis* BCG [34,35].

## Conclusions

This study aimed to further characterize the morphological variations and behaviours exhibited within clinical *M. abscessus* infections. We revealed a relationship between clinical and reference strain behaviour in both phenotypic morphotype and intracellular behaviour.

We highlight the complexity of working with clinical isolates, particularly with the heterogeneity of intracellular replication observed even within each morphotype group. By evaluating strain pairs in an intracellular model, we observed an enhanced growth rate in R compared to S, which likely contributed in part to R strain variability. Together, these data encourage the development of specific R and S strain methodologies when assessing host–pathogen relationships.

Considering these findings, we suggested that the existing S-to-R framework should be considered more as a reference point rather than an absolute standard. This framework can serve as a foundation to drive deeper comprehension of infection progression, as well as the development of virulence and resistance mechanisms. Embracing a more flexible approach to morphotype classification could facilitate a more nuanced understanding of *M. abscessus* infections and aid in the development of more effective therapeutic interventions.

## Supplementary material

10.1099/jmm.0.001869 Uncited Supplementary Material 1.

## Abbreviations

BCCDC: B.C. Centre for Disease Control
CF: cystic fibrosis
c.f.u.: colony-forming units
CO_2_: carbon dioxide
DAT: diacyltrehalose
DPBS: Dulbecco’s phosphate-buffered saline
GPLs: glycopeptidolipids
7H10OADCKan: 7H10 agar supplemented with 0.5 % glycerol, 10 % OADC, and 50 µg ml^−1^ kanamycin
7H9OADCT: 7H9 broth supplemented with 10 OADC and 0.05 % Tween 80
LB: Luria-Bertani
Mab: *Mycobacterium abscessus abscessus*
MABS: *Mycobacterium abscessus*
MAC: *Mycobacterium avium* complex
MALDI-TOF MS: matrix-assisted laser desorption/ionization–time-of-flight mass spectrometry
Mbo: *Mycobacterium abscessus bolletii*
Mma: *Mycobacterium abscessus massiliense*
MOI: multiplicity of infection
NTM: non-tuberculous mycobacteria
PIM: phosphatidylmyo-inositol mannoside
PIM2: phosphatidyl-myo-inositol dimannoside
R: rough
ROI: region of interest
RPMI1640: Roswell Park Memorial Institute medium
S: smooth
TLC: thin layer chromatography
TLR-2: toll-like receptor 2
TMM: trehalose monomycolate
TNTs: tunnelling nanotubes
