# Supplementary material for: The diversity of clinical Mycobacterium abscessus isolates in morphology, glycopeptidolipids and infection rates in a macrophage model
Source: J Med Microbiol. 2024 Aug 19;73(8):001869. doi: 10.1099/jmm.0.001869 (PMC12453391; doi:10.1099/jmm.0.001869)
Supplement: Uncited Supplementary Material 1. [file jmm-73-01869-s001.pdf]

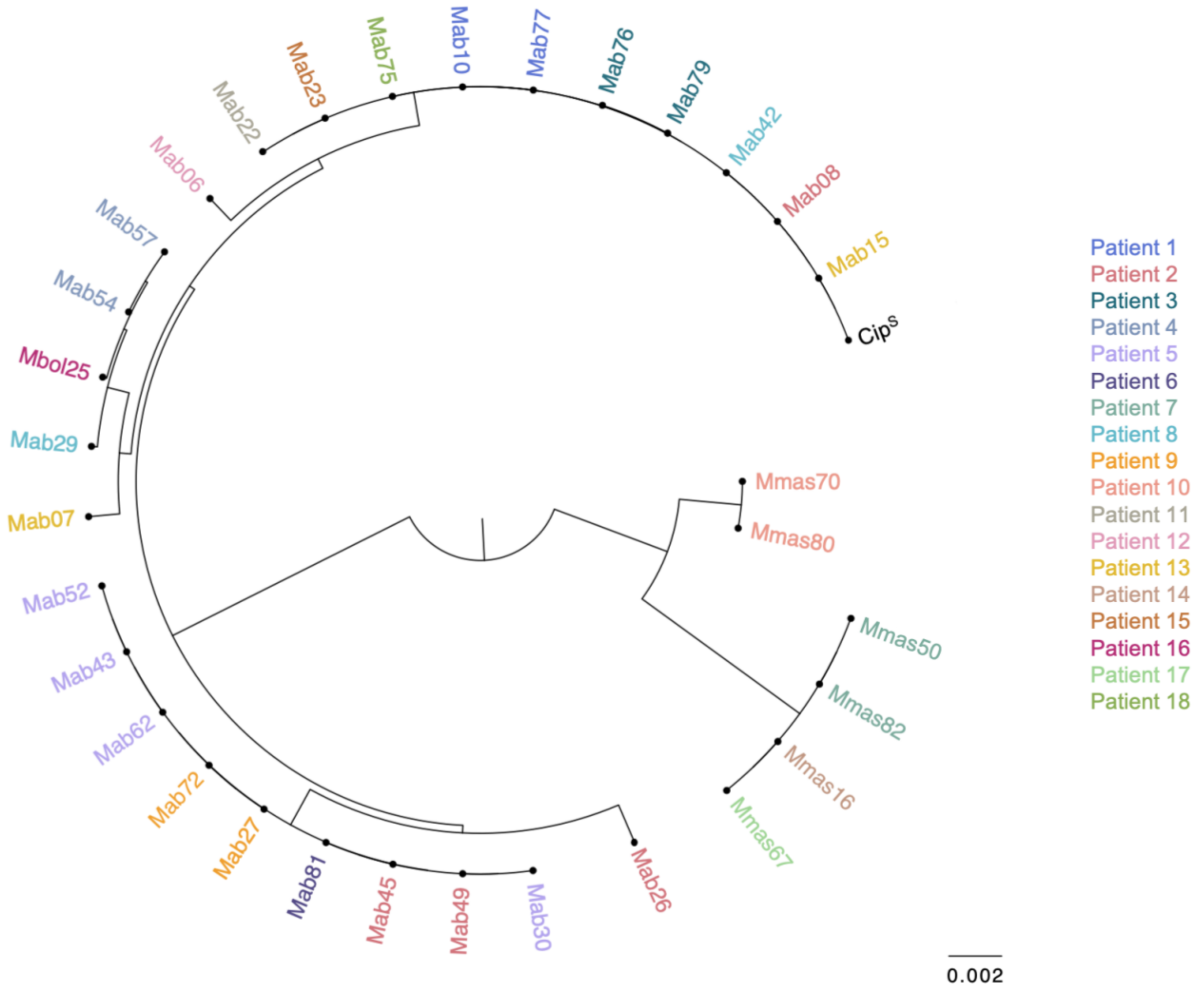

Fig. S1. Phylogenetic tree of unseparated isolates with numeric IDs. Serial isolates from the same patient are represented by the same colour and are clustered, with the exception of isolates from Patients 2, 8 and 13. Clustered serial isolates indicate a likelihood of ongoing infection, rather than co-infection or re-infection. Isolates with alphabetic IDs were received after sequencing was undertaken and are, therefore, absent from the tree. Phylogenetic tree made in FigTree v.1.4.4.

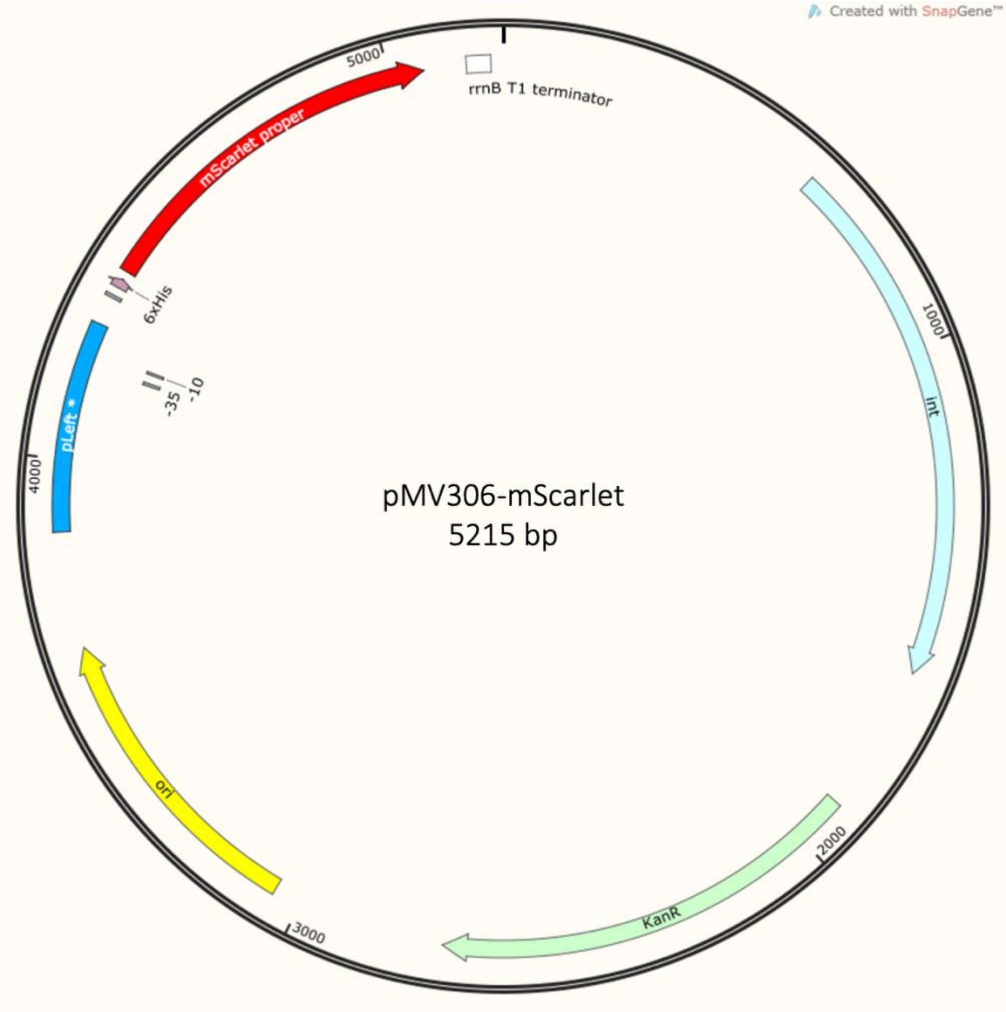

Fig. S2. Construct map of the pMV306-mScarlet plasmid. Derivative versions of the integrative plasmid pMV306 were constructed for strong expression of mScarlet fluorescent proteins with a kanamycin resistant cassette for selection. This plasmid contains pMV306 integrase attP as a one-step integration construct. Plasmid map made in SnapGene v.6.2.0.

Strain	24hr	48hr	72hr
6S	0.44	2.65	3.90
8S	2.22	11.31	16.1
10S	0.29	0.28	2.30
15S	0.65	3.58	5.88
22S	0.41	0.48	3.09
25S	0.68	3.11	27.81
27S	0.85	4.66	6.24
29S	0.16	2.02	2.60
30S	0.41	1.93	6.80
42S	1.02	5.34	12.95
45S	0.23	1.96	7.27
49S	0.39	0.84	4.46
50S	0.71	5.09	21.11
52S	0.20	2.61	11.69
54S	0.37	2.68	4.80
57S	0.21	0.07	0.62
62S	0.28	0.55	7.01
75S	0.65	3.27	6.87
76S	0.55	0.19	1.22
77S	0.76	3.16	6.44
80S	0.17	0.72	4.59
81S	0.50	0.61	4.05
A.S.	0.31	1.65	2.07
B.S.	0.25	0.17	1.51
C.S.	0.17	0.99	3.94
E.S.	0.85	0.14	1.87
F.S.	0.59	0.50	6.67
H.S.	0.37	0.68	8.42
K.S.	0.68	4.40	5.43
L.S.	0.48	0.87	7.51
M.S.	0.10	1.45	8.35
Cip^S	0.36	1.38	19.46
Clinical S average	0.51	2.19	6.89
82R-	0.85	3.14	7.68
6R	0.55	2.24	6.11
23R	0.59	1.13	6.93
30R	0.40	3.19	17.67
42R	0.21	3.29	18.69
43R	0.58	4.90	17.38
45R	0.18	0.96	3.40
52R	0.52	4.00	11.66

54R	0.19	0.54	2.15
57R	0.17	0.86	6.13
62R	1.40	8.46	22.07
75R	1.37	11.41	13.14
77R	1.65	8.92	20.05
81R	0.03	2.43	8.94
82R	0.40	0.63	4.56
A.R.	1.10	6.55	14.16
E.R.	0.74	4.03	4.57
F.R.	0.14	0.86	3.90
G.R.	0.32	3.70	21.94
J.R.	0.22	3.88	18.77
K.R.	0.42	3.93	16.55
M.R.	0.28	4.66	21.64
N.R.	1.37	9.38	20.21
Cip^R	0.12	0.34	3.85
Clinical R average	0.58	3.93	12.37

Table. S1. Fold-changes values of each strain at 24, 48 and 72 hours. Values were generated in triplicate by capturing relative fluorescence units (RFU) with a high-content fluorescent microscope (CellInsightTM CX5 High Content platform, Thermo Fisher Scientific) and normalised to the 4-hour post-infection values. At the final time point of 72 hours, the reference strain Cip^S grew ~2x more than the Cip^R, while inversely, the average clinical smooth (S) strains grew ~2x less than the average clinical rough (R) strains. Some isogenic pairs showed the S replicating faster than the R (isolates F and 54 of Patients 1 and 4, respectively), which was then reversed in the subsequent serial isogenic pairs (isolates 77 and 57, respectively). In both instances, this was accompanied by the decline in lung function capacity (data not shown), highlighting the variability that exists among clinical strains even within the same infection.
